# Analysis of hydraulic losses in the series from 1990 to 2022 at the Retiro small hydroelectric power plant

**DOI:** 10.1016/j.heliyon.2024.e29627

**Published:** 2024-04-16

**Authors:** Francisco Wellington Martins da Silva, José Roberto Camacho, Jacson Hudson Inácio Ferreira

**Affiliations:** a Laboratory of Alternative Energies and Power System Protection, Universidade Federal de Uberlândia, 2121 João Naves de Ávila Ave., Bldg. 5K, Campus Santa Mônica, Uberlândia, Minas Gerais, 38400-902, Brazil; b Instituto Federal do Triângulo Mineiro, Campus Ituiutaba, Belarmino Vilela Junqueira St., No number, Novo Tempo 2, Ituiutaba, Minas Gerais, 38305-200, Brazil

**Keywords:** Average Power, Efficiency, Energy, Head Losses, Small Hydroelectric Power Plant, Statistical Analysis

## Abstract

Hydraulic losses are a crucial variable in hydroelectric ventures as they can cause significant reductions in power generation. This article analyses the impact of hydraulic losses on the Retiro Small Hydroelectric Power Plant (SHPP), investigating their effect on monthly average power and, consequently, the efficiency of electricity generation. This study examines the historical series of load losses at the Retiro SHPP from 1990 to 2022. The calculations are based on flow data available in HidroWeb. The study considered the maximum and minimum flow rates in the historical flow series as constraints for hydropower generation. We used a multivariable function to calculate the efficiency of the hydraulic turbine, relating the turbine flow rate and the net water head. We developed mathematical relationships for head losses that occur in the grid, at the water intake, and due to friction from the intake to the turbine of the Retiro SHPP. The article presents a comparison between the actual monthly average power of the Small Hydroelectric Power Plant (SHPP) and the simulated monthly average power. We normalized the data for turbine flow and load loss to conduct statistical analysis. Kernel probability density was applied to understand the distribution shape of the data. Findings show that average monthly capacity is lowest in September, at approximately 0.81 MW. In March, the highest power occurs, approximately 14.19 MW. During the high flow period, the simulated average power, which accounts for load losses, closely matched the actual average power generated at the Retiro SHPP. In the months from July to October, despite being the period with the lowest head losses, it is the time when there is a greater opportunity to maximize energy generation at the power plant. An inefficient power generation system experiences significant load losses during specific periods of the year. To minimize this effect, it is crucial to understand the behavior of hydraulic losses and consider implementing mitigating measures.

## 1 Introduction

Energy plays a crucial role in shaping the development trajectory of nations and in defining the living standards of their populations. Analysts consider the generation and consumption of electrical energy as variables when classifying a country as developed [1]. Due to rapid population growth and industrialization worldwide, the demand for energy is increasing swiftly [2].

Electricity production in Brazil primarily derives from large hydroelectric power plants located along rivers with significant water volumes. As sites with greater hydropower potential are developed and competing water uses increase, the system expansion becomes more expensive and challenging [3]. Although Brazil has already tapped into its hydroelectric potential, there is still much to be explored, especially in small rivers that are tributaries to larger rivers where large hydroelectric plants are installed.

Hydroelectric power plants are among the most efficient and reliable renewable energy systems in the world when it comes to producing electricity. The use of renewable energies from water sources corresponds to a considerable percentage of the energy matrix in Brazil. According to ANEEL's (National Electric Energy Agency) Generation Information System (SIGA) [4], approximately 53.44 % of energy generation capacity comes from Hydroelectric Power Plants (HPPs), which represents, on average, 103,487,521 kW, about 2.97 % comes from Small Hydroelectric Power Plants (SHPPs) and 0.46 % from very small Hydroelectric Generating Plants (HGPs). These numbers show that Brazil is one of the leading countries in the world regarding hydroelectrical participation in the total production of electric energy [5].

Over the years, the demand for energy has been driving projects and studies on new, cleaner, and environmentally friendly potential energy sources. This has stimulated an increase in the demand for Small Hydroelectric Power Plants (SHPPs) [6]. Run-of-the-river hydroelectric plants, like SHPPs, seem more attractive than conventional hydroelectric plants, as they can be a cheaper and better ecological alternative [7]. The SHPPs represent small hydroelectric projects. To define those projects normative resolution No. 875 of March 10, 2020, by ANEEL [8] characterizes a SHPP as an undertaking that has an installed capacity between 5 MW and 30 MW and with a reservoir that cannot exceed 13 km^2^. This type of venture causes less flooding of arable areas, and consequently, fewer environmental problems related to erosion, sediment accumulation, and modification of the river's original bed. Its hydro mechanical equipment is smaller and simplifies the logistics of transportation and maintenance.

Small Hydroelectric Power Plants have an inherent capacity for instant start, stop, load shedding, etc., and help improve the reliability of the power system, making them the best choice to meet peak demand [2,9,10]. SHPPs in developing countries, such as Brazil, can become more competitive by trading emission reductions achieved under the Clean Development Mechanism, as result of the Kyoto Protocol proposed in the United Nations Framework Convention on Climate Change [11]. In contrast to fossil fuel-powered plants, such as thermal power plants, SHPPs generate electrical energy with minimal carbon emissions. Hydroelectric plants help reduce greenhouse gas emissions, expand renewable energy generation, and distribute locally the national energy generation, by supplying energy closest to the loads. This is especially true for Small Hydroelectric Power Plants [12].

According to ANEEL's Generation Information System as of mid-2023 [4], Brazil currently has 426 operational SHPPs, with 31 under construction and 77 pending constructions. The total granted power of the operational SHPPs is approximately 5,719,975.57 kW, which could increase to over 7,239,264.22 kW once the project ventures are completed. May exceed 3.5 % of all installed power of the national energy matrix in the coming years. In this context, SHPPs become a strategic source of energy for sustainable development in Brazil.

When analyzing power generation efficiency, it is important to consider the reduction of head losses. Brazil has a significant hydroelectric generation capacity, which means that even small percentage losses in the system can represent significant values. Load losses can mean a considerable portion of the availability of existing free fall potential in the installation and may vary up to 2 % for small drop facilities [13]. Head loss estimation in pressurized pipelines is a common issue in optimization studies, hydraulic analysis of pipelines, and water distribution systems [14]. For the tunnel project, understanding the head loss is an economic issue and one can consider a waived amount of energy [15]. To detect points of head loss, it is crucial to understand the hydraulic circuit that makes up the project.

Several authors have researched load losses in hydroelectric systems. Jardim et al. [16] adapted mathematical models to determine the friction factor that determines load losses. Bastos et al. [17] present a case study estimating the loss of generation caused by an increase in the roughness of the hydraulic circuit of the small hydroelectric plant Repi in the municipality of Wenceslau Braz, Minas Gerais, Brazil. Milukow et al. [18] estimated the Darcy-Weisbach friction factor for unlimited streams using gene expression programming and extreme learning machines. Pimenta [19] analyzes explicit load factor of approximations for pressurized conduits compared to the formulation of Colebrook-White. Moody's [20] algorithm provides estimates for friction factors to be used in calculating load losses in pipes and closed conduits running full flow with constant flow rate. Leite [13] examines the effects of increased head loss on small hydroelectric installations, particularly power-generating plants (CGH) equipped with low- and high-pressure ducts. This is due to the influence of increased internal roughness of conduct adduction and its impact on the reduction of firm energy in these facilities.

The aim of this study is to estimate hydraulic losses in energy generation at the Retiro Small Hydroelectric Plant from 1990 to 2022. The study will focus on the hydraulic circuit configurations and their impact on energy generation. To improve the efficiency of the electrical system, it is important to analyze energy generation variables. In the context of the Brazilian energy matrix, it is important to consider the role of SHPPs as a source that could enable a greater reduction in carbon emissions compared to other conventional energy sources. Therefore, investment policies should be stimulated to modernize SHPPs, create optimal operation methodologies for energy generation, and minimize load losses, among other considerations, to promote and invest in this clean energy source.

## 2 Materials and methods

### 2.1 Data and study object

The methodology applied in this study involves the use of flow data from the historical series of fluviometric stations *Fazenda São Domingos* (61787500) and *São Domingos* (61788000), which are in the hydrological influence region of the Retiro Small Hydroelectric Power Plant (SHPP). Data were obtained from the website: https://www.snirh.gov.br/hidroweb/apresentacao.

SHPP Retiro with an installed capacity of 16 MW is located on the Sapucai River, in the Municipality of Guará in the State of São Paulo in the Rio Grande sub-basin, Brazil. The flood area is approximately 2.96 km^2^, occupying an average water volume of 17.87 hm³. The Small Hydroelectric Power Plant (SHPP) is equipped with a three-phase synchronous generator. The generator has a power output of 17,590 kVA, a nominal voltage of 6900 V, a nominal power factor of 0.9, and operates at a nominal frequency of 60 Hz. Its nominal speed for continuous operation is 600 rpm. The system is driven by a horizontal axis Well Bulb hydraulic turbine with a power output of approximately 16,236 kW. The maximum intake flow of the turbine is 154.5 m³/s and the minimum intake flow corresponds to approximately 21 % of that value. It is the last SHPP on the Sapucai River, where it flows into the Porto Colombia dam, on the border of the State of Minas Gerais and the State of São Paulo. The start of operations for the Retiro Small Hydroelectric Plant occurred around 2014. Conducting studies considering a historical series before the project's operation strengthens the argumentation regarding the generation capacity estimate, as well as confirming whether the feasibility studies were adequately planned.

We used monthly flow data from the historical series from 1990 to 2022 to calculate flow velocity, power, and load losses. For periods in which the influent flow (*Q*_*af*_) is below the minimum turbine flow ($Q_{turb}^{\min}$) of 32.50 m³/s, a flow rate of Q = 0 is considered for the calculation, according to relation (1), and there is no energy generation. In periods where *Q*_*af*_ is above $Q_{turb}^{\min}$ and below the maximum turbine flow ($Q_{turb}^{\max}$), the turbine flow (Q) is equal to the influent flow *Q*_*af*_ as per relation (2). In periods where Qaf exceeds the maximum turbine flow ($Q_{turb}^{\max}$) of 154.5 m³/s, the flow considered for this calculation follows relation (3), and the excess flow is discharged through the spillway.

(1) $$\text{If}\;Q_{af}\;<\;Q_{turb}^{\min}\;\text{then}\;Q=0$$

(2) $$\text{If}\;Q_{turb}^{\min}\;<\;Q_{af}\leq Q_{turb}^{\max}\;\text{then}\;Q=Q_{af}$$

(3) $$\text{If}\;Q_{af}\;>\;Q_{turb}^{\max}\;\text{Then}\;Q={\;Q}_{turb}^{\max}=154,5\;m^{3}/s$$

The equation for *Q*_*af*_ is based on the inflow (m³/s), while $Q_{turb}^{\max}$ and $Q_{turb}^{\min}$ represent the maximum and minimum turbine flow (m³/s), respectively. Q refers to the flow passing through the turbine (m³/s).

We conducted the estimation of head loss behavior over the months of the historical series. This calculation was performed in accordance with the hydraulic circuit settings of the Retiro Small Hydroelectric Power Plant (SHPP). Afterward, we conducted the power generation calculation and compared it to the real power data provided by the unit operator. We normalized the flow and head loss data for pattern analysis. We then applied the statistical technique of probability density using the Kernel function to these data.

**Fig. 1:**
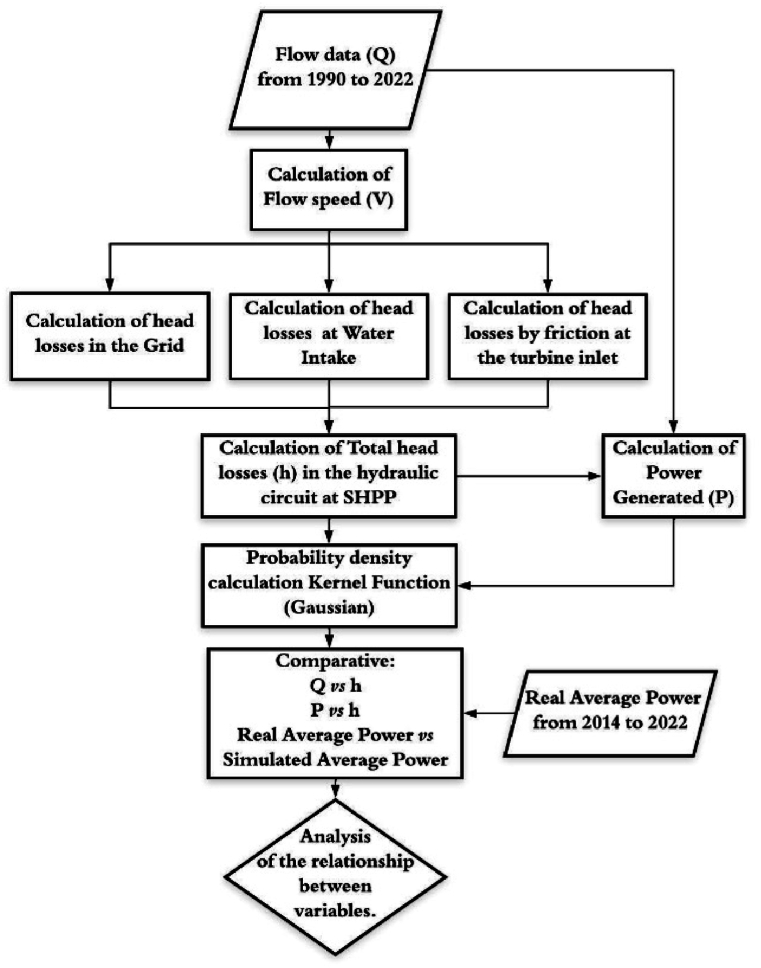
Flowchart of the adopted methodology.

The flowchart in Fig. 1 summarizes the methodology adopted in this study.

### 2.2 Calculation of the pressure losses for each component of the hydraulic circuit in the Retiro Small Hydroelectric Plant

The Retiro Small Hydroelectric Power Plant (SHPP) does not have a conduit and/or tunnel in its civil structure. This study considers the following: head losses in the grid, head losses at the water intake entrance, head losses due to friction at the entrance to the turbine in the powerhouse. To replicate this study in other Small Hydroelectric Power Plants (SHPPs), it is crucial to understand the specific characteristics of the hydraulic circuit of the respective SHPP, also examining other points of head loss, such as the intake channel, inlets, among other factors. Table 1 presents the main characteristics of the hydraulic circuit considered for this study. The National Electric Energy Agency (ANEEL) provided the data obtained from the SHPP Retiro base project.

**Table 1:** Characteristics of the hydraulic circuit elements of SHPP Retiro.

WATER INLET	
Upstream gate section area (*A*_*1*_)	93.54 m^2^
Base of the upstream gate (*b*)	8.8 m
Upstream gate height (*h*)	10.63 m
**GRIDS**	
Grids area (*A*_*2*_)	135.73 m^2^
Bar thickness (*e*_*1*_)	1.00 cm
Bar width (*e*_*2*_)	6.00 cm
Head loss coefficient (*k*_*g*_)	2.42
Grid tilt (*φ*)	78.89°
**WATER OUTLET INPUT**	
Head Loss Coefficient (*k*_*e*_)	0.15
**ENTRANCE TO THE TURBINE**	
Pipe length *(L*)	24.24 m
Internal diameter of the pipe (base or height of the arc-rectangle section) (*D*)	10.91 m

In a SHPP, attention deserves the variable of head loss with its relation to the efficiency in energy generation. The smaller the head losses, the greater the energy generation. Therefore, it is extremely important to ensure that the system operates with the lowest possible pressure drop. This identifies the opportunity to optimize power generation.

Equations (4), (5)), respectively can determine the flow speed of water in the upstream gate section and the grid section [21].

(4) $$V_{1}=\frac{Q}{A_{1}}$$

(5) $$V_{2}=\frac{Q}{A_{2}}$$

Where $V_{1}$ is the flow speed (m/s) in the upstream gate section, $V_{2\;}$ is the flow speed (m/s) in the grids section, $A_{1\;}$ is the area of the upstream gate (m^2^), $A_{2\;}$ is the area of the grills (m^2^) and Q is the flow (m³/s).

The grid, on the upstream face of the water intake, aims to prevent the entry of floating bodies that could damage the equipment [15]. The act of containment of the debris at the entrance of the equipment can interrupt the flow of water and generate load losses. Equation (6) can calculate these grid losses [13,15].

(6) $${\Delta h}_{g}=k_{g}{\left(\frac{e_{1}}{e_{2}}\right)}^{\frac{4}{3}}.\sin\;\varphi \frac{V_{2}^{2}}{2g}$$

Where Δ *h*_*g*_ is the pressure drop on the grill (m), *k*_*g*_ head loss coefficient that depends on the dimensions of the grid, *e*_*1*_ is the thickness of the bar (cm), *e*_*2*_ is the width of the bar (cm), *φ* is the grid inclination in degrees and *g* is the acceleration due to gravity (m/s^2^).

According to ELETROBRÁS [15], the determination of the head loss coefficient is given by:

$$If\;\frac{e_{1}}{b}\geq 5$$

For rectangular bars, $k_{g}=2.42$.

For circular bars, $k_{g}=1.79$.

The water intake is the work intended to capture water necessary for the operation of the hydraulic turbines. It must contain devices to eliminate or retain solid material carried by water that could damage the turbines [22]. Equation (7) defines the head loss due to entering the water intake.

(7) $${\Delta h}_{e}=k_{e}\frac{V_{1}^{2}}{2g}$$

where *k*_*e*_ is the head loss coefficient in the water intake.

Equation (7) of Darcy-Weisbach is of frequent use in research to demonstrate energy losses in the generation system due to attrition/friction of the water on the pipe walls [23,24]. This research uses Equation (8) to calculate the head loss due to friction at the turbine inlet.

(8) $${\Delta h}_{t}=k_{f}∙\frac{L}{D}∙\frac{V_{1}^{2}}{2g}$$

Where ${\Delta h}_{t}$ is the head loss (m), $k_{f}$ is the friction head loss coefficient, *L* is the length of the pipe (m), *D* is the reference diameter (base or height of the arc-rectangle section) (m), $V_{1}$ is the speed of water flow in the cross section of the pipe (m/s) and *g* is the acceleration due to gravity (m/s^2^).

The head loss coefficient $k_{f}$ is a ratio of wall roughness, tunnel reference diameter and flow speed. It can be calculated using Equation (9).

(9) $$k_{f}=124,58\frac{n^{2}}{D^{0,333}}$$

where, *n* is the Manning coefficient, which varies depending on the roughness of the tunnel walls (in relation to the type of coating).

For this study, according to the Manuals and Guidelines for Projects of Small Hydroelectric Power Plants [15], for the concrete-type coating, the Manning coefficient to be adopted is *n* = 0.013. For other types of coatings, it is possible to obtain other coefficients in Mataix [25].

The definition of total head loss in Equation (10) for the hydraulic circuit is as the sum of the partial head losses from Equation (11), (12), (13)).

(10) $${\Delta h}_{f}={\Delta h}_{g}+{\Delta h}_{et}+{\Delta h}_{t}$$

(11) $${\Delta h}_{f}=k_{g}{\left(\frac{e_{1}}{e_{2}}\right)}^{\frac{4}{3}}.\sin\;\varphi \frac{V_{2}^{2}}{2g}+k_{e}\frac{V_{1}^{2}}{2g}+k_{f}∙\frac{L}{D}∙\frac{V_{1}^{2}}{2g}$$

(12) $${\Delta h}_{f}=\frac{k_{g}{\left(\frac{e_{1}}{e_{2}}\right)}^{\frac{4}{3}}.\sin\;\varphi V_{2}^{2}}{2g}+\frac{124,58k_{e}{V_{1}^{4}n}^{2}L}{{{4g}^{2}D}^{1,333}}$$

(13) $${\Delta h}_{f}=\frac{k_{g}{\left(\frac{e_{1}}{e_{2}}\right)}^{\frac{4}{3}}.\sin\;\varphi {\left(\frac{Q}{A_{2\;}}\right)}^{2}}{2g}+\frac{124,58k_{e}{{\left(\frac{Q}{A_{1\;}}\right)}^{4}n}^{2}L}{{{4g}^{2}D}^{1,333}}$$

### 2.3 Normalization of flow and head loss data

For a comparison on the same scale, we performed a normalization of the Flow and Head Loss data according to Equation (14).

(14) $$z=\frac{x-\bar{x}}{\sigma }$$

Where z represents the standardized variable, *x* represents the monthly flow and/or head loss variable of the historical series, $\bar{x}$ represents the monthly average flow and/or head loss variable of the historical series, and σ represents the monthly standard deviation of the series.

### 2.4 Calculation of the net drop height of SHPP retiro for the 32 years (1990–2022) series

Table 2 presents a relation between flow and downstream water level provided by ANEEL. Measurements taken at the project during different periods and operational conditions provided these data. Subsequently, we organized them in ascending order of flow rate. We can obtain a tailrace discharge curve, adjust it and thus find a relationship for calculating the net height of the SHPP Retiro for the studied series.

**Table 2:** Flow vs Quota of measured water level.

Flow (m^3^/s)	Downstream level (m)
29.1	509.16
34.1	509.20
38.2	509.24
42.1	509.27
45.4	509.29
48.6	509.31
52.0	509.34
56.5	509.37
61.6	509.41
67.9	509.46
74.1	509.50
80.9	509.55
91.0	509.62
99.1	509.67
110.4	509.75
122.0	509.82
138.7	509.93
158.3	510.04
182.1	510.18
358.5	510.88

The base curve was drawn plotted using the data from Table 2. This methodology allows understanding how the flow rate varies as a function of the water downstream level. Observing patterns and trends is essential for comprehending the hydraulic behavior of the system. Aiming to find the coefficients of the polynomial that minimize the sum of squares of the residuals between measured values and predicted values. The Least Squares Method (LSM) is a procedure to determine the coefficients for the best fit to the data. Griffiths and Smith [26,27] describes the LSM procedure.

Equation (15) describes the parameters of the second-order equation, obtained through the Least Squares Method (LSM) during the fitting of the key curve. This equation relates the flow level of the Small Hydroelectric Power Plant (SHPP) in terms of elevation, referenced to sea level.

(15) $$f\left(x\right)=-7.637∙10^{-6}x^{2}+0.008158\;x+508.9$$

**Fig. 2:**
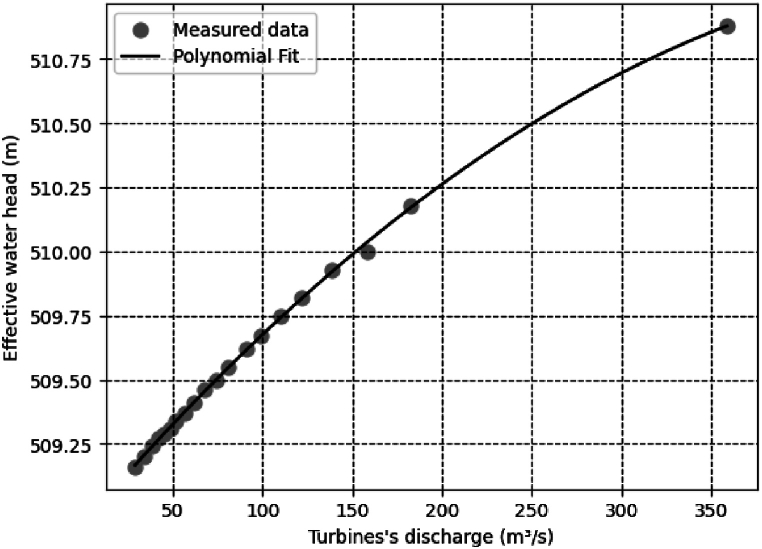
Tailgate discharge key curve.

From the data in Table 2, a representative key curve is drawn. Fig. 2 graphically shows the result of 20 flow measurements for SHPP Retiro. Each point on the graph corresponds to a flow measurement and corresponding to water level. The solid line represents the least squares-fitted key curve.

Through the polynomial function depicted in Equation (15), we got a determination coefficient (R^2^) of 0.9994, which indicates that the adjusted key curve represents most of the variability of the measured data. Which characterizes most of the gross fall data for the historical series of SHPP Retiro. For another more assertive analysis, considering another historical series, this suggests building another discharge characteristic curve.

Equation (15) calculates the gross head. Therefore, to obtain the value of the gross head (*h*) for the Retiro Small Hydroelectric Power Plant (SHPP), the value obtained in Equation (15) is subtracted from the maximum normal upstream level (${NA}_{up}$) relative to sea level. Thus, Equation (16) determines the gross head of the Retiro SHPP.

(16) $$h={NA}_{up}-\;\left(-7.637.10^{-6}Q^{2}+0.008158\;Q+508.9\right)$$

Where *h* represents the gross drop height of the SHPP, *Q* the outflow of the historical series considered in this study and ${NA}_{up}$ is the maximum normal upstream level.

According to the base project approved by ANEEL, the maximum normal upstream level (${NA}_{up}$) for the Retiro Small Hydroelectric Power Plant is 522.5 m relative to sea level.

The net head (*H*_*t*_), according to Equation (17), is determined by the difference between the gross head (*h*), given by Equation (16), and the total head loss (${\Delta h}_{f}$), given by Equation (13).

(17) $${H_{t}=h-\Delta h}_{f}$$

### 2.5 Calculation of power generated by SHPP retiro for the 32-year series (1990–2022)

This study proposes to use the methodology presented by the Energy Research Company EPE to represent the hill curve to obtain hydraulic turbine performance [28]. The data to derive the function include net head and its corresponding flow rate for Table 3. We apply the least squares technique to obtain the coefficients of the function based on these points.

**Table 3:** Turbine Test Data presented in the project.

Turbine flow (m³/s)	Net drop (m)	Turbine efficiency (%)
154.5	10.69	91.44
154.5	11.59	92.24
154.5	11.73	92.38
136.7	12.00	92.77
120.0	12.24	92.74
108.4	12.38	92.44
97.1	12.54	92.14
89.0	12.54	91.74
78.9	12.75	90.75
72.4	12.84	89.55
65.9	12.91	88.37
59.6	12.92	85.82
54.5	13.04	85.39
50.0	13.08	83.99
46.6	13.12	82.85
43.4	13.15	81.71
40.1	13.18	80.45
36.2	13.22	78.85

The hill curve can be represented, analytically, by a second-degree polynomial as a function of the turbine flow (*Q)* and the net height of the waterfall (*H*_*t*_) [[28], [29], [30], [31], [32]]. Using least-squares regression, we obtained Equation (18) as the fitted multivariable, second-degree best-fit polynomial. The polynomial coefficients represent the numerical parameters and $\eta_{t}$ represents the turbine efficiency as a function of the net head and turbine flow.

(18) $$\eta_{t}=-6.5576∙10^{-5}Q^{2}-0.097{4H}_{t}^{2}-0.00491H_{t}Q+0.0754Q+2.9449H_{t}-21.4012$$

The $P_{G}$ function represents the power generated by the SHPP. The power is dependent on the productivity of the system and the turbine flow (*Q*) [33]. The productivity of the system is the product of the respective turbine efficiency $\left(\eta_{t}\right)$, Generator efficiency ($\eta_{g})$ which is 0.98, the specific weight of the water (ρ), the acceleration of gravity (g) and the net height ($H_{t})$ of the upstream water level.

(19) $$P_{G}=\eta_{t}\eta_{g}{\rho gH}_{t}Q$$

## 3 Results and discussion

Table 4 shows the head losses of the SHPP of Retiro from the 32 years (1990–2022) in the historical series obtained from relation 13. There may be a seasonal trend in hydraulic losses; similar observations are made in flow rate data. We believe strongly that variations in water flow, upstream water level in the reservoir and seasonal operation criteria of the installation is related to this effect.

**Table 4:** Annual and monthly average of the head loss historical series.

Year	JAN	FEB	MAR	APR	MAY	JUN	JUL	AUG	SEP	OCT	NOV	DEC	Average Annual
**1990**	0.038	0.033	0.038	0.031	0.021	0.013	0.009	0.008	0.006	0.008	0.006	0.007	**0.018**
**1991**	0.037	0.038	0.038	0.038	0.038	0.027	0.018	0.011	0.006	0.007	0.006	0.013	**0.023**
**1992**	0.038	0.038	0.038	0.038	0.034	0.016	0.011	0.007	0.010	0.021	0.034	0.024	**0.026**
**1993**	0.022	0.038	0.038	0.038	0.025	0.019	0.009	0.006	0.006	0.006	0.003	0.009	**0.018**
**1994**	0.038	0.029	0.038	0.021	0.014	0.008	0.005	0.003	0.002	0.003	0.007	0.017	**0.015**
**1995**	0.023	0.038	0.023	0.020	0.016	0.010	0.011	0.005	0.004	0.006	0.006	0.020	**0.015**
**1996**	0.038	0.038	0.038	0.032	0.019	0.009	0.007	0.005	0.006	0.004	0.018	0.026	**0.020**
**1997**	0.038	0.038	0.038	0.035	0.022	0.023	0.010	0.006	0.004	0.004	0.007	0.025	**0.021**
**1998**	0.018	0.038	0.038	0.026	0.020	0.011	0.005	0.004	0.002	0.005	0.009	0.014	**0.016**
**1999**	0.038	0.038	0.038	0.027	0.013	0.009	0.005	0.003	0.003	0.000	0.002	0.009	**0.016**
**2000**	0.038	0.038	0.038	0.034	0.014	0.008	0.005	0.004	0.006	0.002	0.003	0.007	**0.016**
**2001**	0.006	0.006	0.005	0.004	0.002	0.000	0.000	0.000	0.000	0.000	0.002	0.017	**0.004**
**2002**	0.034	0.038	0.038	0.020	0.011	0.005	0.004	0.002	0.002	0.000	0.004	0.010	**0.014**
**2003**	0.038	0.038	0.038	0.038	0.028	0.013	0.009	0.006	0.003	0.003	0.004	0.008	**0.019**
**2004**	0.017	0.038	0.038	0.038	0.027	0.017	0.011	0.006	0.004	0.006	0.005	0.026	**0.019**
**2005**	0.038	0.038	0.037	0.017	0.019	0.012	0.007	0.004	0.003	0.002	0.005	0.023	**0.017**
**2006**	0.038	0.038	0.038	0.027	0.012	0.007	0.004	0.003	0.002	0.005	0.006	0.013	**0.016**
**2007**	0.032	0.038	0.038	0.026	0.015	0.010	0.004	0.005	0.003	0.003	0.005	0.008	**0.016**
**2008**	0.014	0.038	0.038	0.038	0.037	0.020	0.010	0.006	0.004	0.004	0.004	0.016	**0.019**
**2009**	0.038	0.038	0.038	0.038	0.032	0.017	0.011	0.007	0.010	0.010	0.008	0.038	**0.024**
**2010**	0.038	0.038	0.038	0.017	0.011	0.008	0.005	0.003	0.002	0.005	0.008	0.010	**0.015**
**2011**	0.038	0.024	0.038	0.038	0.026	0.015	0.007	0.005	0.003	0.004	0.004	0.009	**0.018**
**2012**	0.038	0.031	0.017	0.014	0.009	0.008	0.005	0.003	0.002	0.002	0.003	0.005	**0.011**
**2013**	0.016	0.037	0.038	0.038	0.018	0.029	0.007	0.004	0.003	0.004	0.007	0.012	**0.018**
**2014**	0.010	0.005	0.005	0.005	0.003	0.002	0.000	0.000	0.000	0.000	0.000	0.008	**0.003**
**2015**	0.003	0.008	0.013	0.015	0.011	0.006	0.004	0.002	0.003	0.000	0.005	0.018	**0.007**
**2016**	0.038	0.038	0.038	0.027	0.018	0.015	0.006	0.004	0.003	0.003	0.006	0.007	**0.017**
**2017**	0.019	0.029	0.018	0.010	0.008	0.004	0.002	0.000	0.000	0.000	0.003	0.014	**0.009**
**2018**	0.038	0.024	0.038	0.012	0.009	0.004	0.003	0.002	0.000	0.004	0.024	0.032	**0.016**
**2019**	0.018	0.036	0.038	0.038	0.026	0.012	0.003	0.000	0.002	0.003	0.007	0.009	**0.016**
**2020**	0.024	0.038	0.038	0.022	0.010	0.006	0.004	0.002	0.002	0.003	0.007	0.010	**0.014**
**2021**	0.011	0.004	0.009	0.004	0.010	0.007	0.000	0.000	0.000	0.005	0.007	0.007	**0.005**
**2022**	0.028	0.038	0.038	0.022	0.011	0.005	0.002	0.000	0.002	0.005	0.002	0.020	**0.015**
**Average Monthly**	**0.029**	**0.032**	**0.033**	**0.026**	**0.018**	**0.011**	**0.006**	**0.004**	**0.003**	**0.004**	**0.007**	**0.015**	

**Fig. 3:**
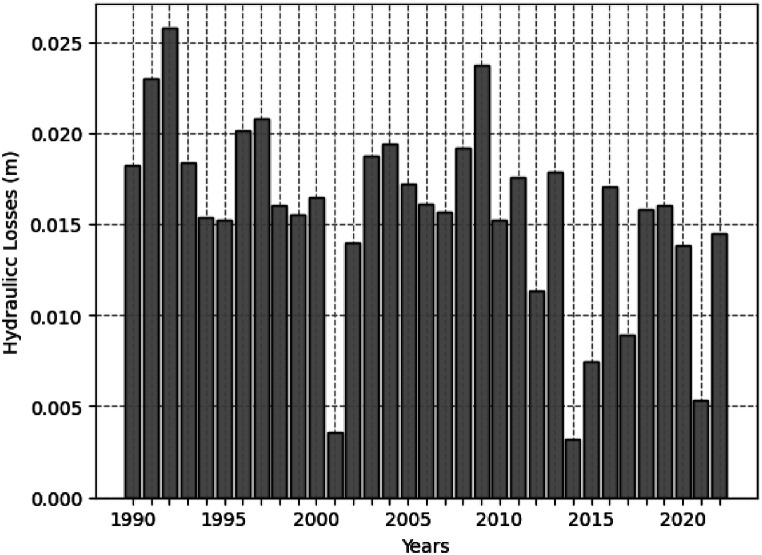
Average annual hydraulic losses in the 32-year historical series (1990–2022).

Fig. 3 represents the series of average annual head losses. In 1992, there was the highest average head loss of the studied series, around 0.026 m. While in 2014, the lowest head loss recorded is around 0.003 m. In the year 2001, the low head loss value observed, is slightly over 0.004 m. There is a downward trend in head loss throughout the studied series, possibly due to a decrease in the average river flow over the recent years.

**Fig. 4:**
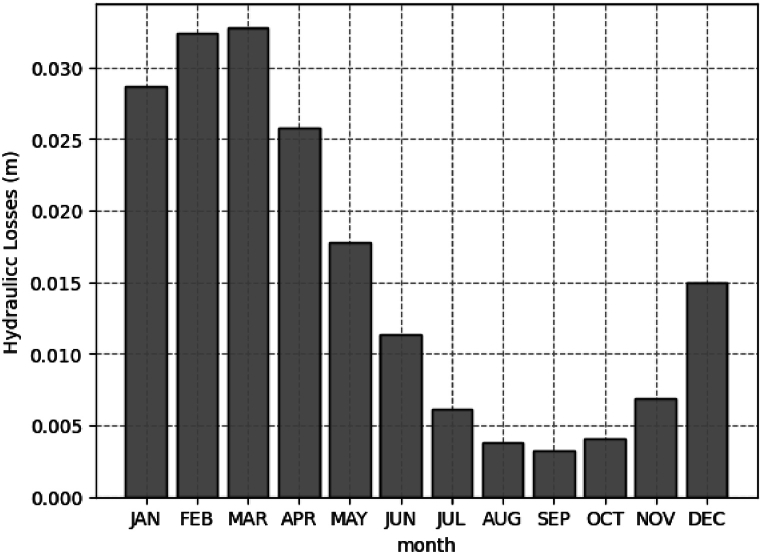
Monthly average hydraulic losses in the 32-year historical series (1990–2022).

Fig. 4 represents the average monthly head losses of the studied series. It is observed that the first quarter of the year had the highest total head losses. In March, it presented, on average, 0.033 m of head losses. In the months of August, September and October the lowest head losses occur, respectively 0.0038 m, 0.0032 m and 0.0040 m. Then the head loss slightly increases towards the end of the year with the resumption of the rainy season. The trend of decreasing head losses follows the climatic behavior. In this specific case, higher water availability resulted in higher losses, while water scarcity led to a more efficient system with lower head losses.

**Fig. 5:**
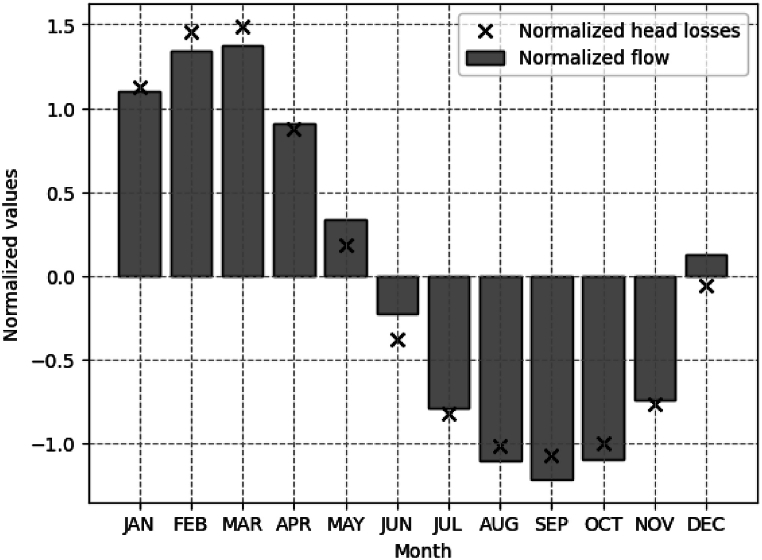
Normalized monthly average hydraulic head losses in the 32-year historical series (1990–2022).

Fig. 5 displays the relationship between the normalized monthly head loss values obtained from Table 4 and the normalized monthly flow data from the historical series spanning from 1990 to 2022. A consistent trend is observed throughout the months. For the first five months of the year, the standardized head loss values are above average, with respective values of 1.12, 1.45, 1.4, 0.87, and 0.17 for January, February, March, April, and May. The data shows that in December, the normalized flow values are above average (0.12), while the normalized head loss data are below average (−0.06). From June to November, standardized values were below average. This suggests that there is a greater head loss in energy production during the first four months of the year for the SHPP Retiro.

**Fig. 6:**
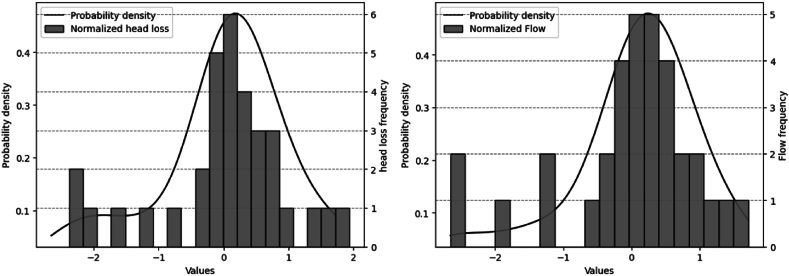
a) Relationship between probability density and flow frequency. b) Relationship between probability density and head loss frequency.

The graph in Fig. 6 a) shows the relationship between the probability density (Gaussian Kernel Function) and the frequency of the normalized annual flow data. The Kernel function suggests that there is a greater number of discharge values above the average in a more dispersed way. While there are fewer values below the mean, with values concentrated between −1 and 0. The probability density plot suggests that most of the data revolve around the mean. The graph in Fig. 6 b) presents the relationship between the probability density (Gaussian Kernel Function) and the frequency of the normalized annual head loss data obtained from the data in Table 4. There are more values above the average and fewer values below the average. This suggests that in most of the historical series the head loss data contribute with significant losses in energy generation. The relationship between head loss and energy becomes more evident.

**Fig. 7:**
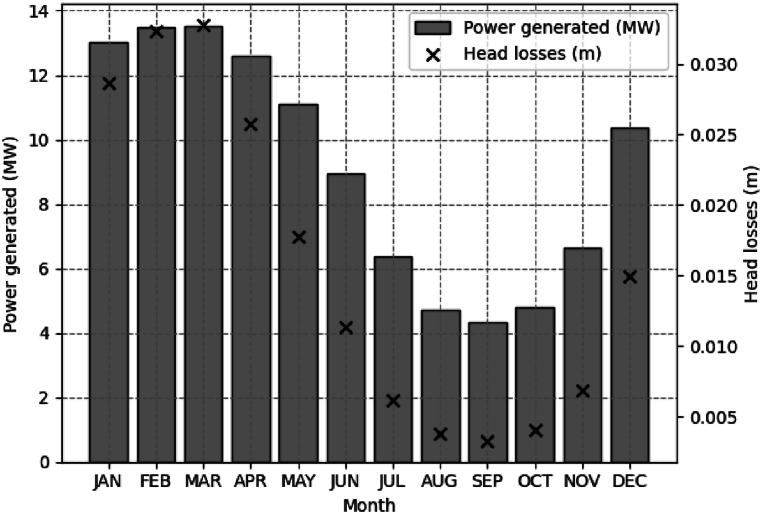
Relation between the average power generated and head losses in the 32-years historical series (1990–2022).

Fig. 7 illustrates the relationship between the average power of the studied series and the average head losses. The bar graph displays the average power with reference values on the left side, while the dot graph shows the head losses with reference values on the right side.

The first four months are characterized as the period of the highest energy production, as they present the highest powers production. This may be related to factors such as a higher volume of water due to seasonal rains. During this same period, there were also higher head losses, particularly in February and March, which exhibited a head loss of approximately 0.03 m. From May to June, energy production remains relatively high but gradually decreases. The graph shows a gradual reduction in average power from July to September, following the same trend as head losses. August, September, and October had the lowest average powers, slightly less than 4.5 MW, which is almost four times lower than the maximum potential the SHPP could achieve, along with the lowest head losses. The observed pattern in the series suggests that head losses have a direct impact on generated power conditions in most months.

**Fig. 8:**
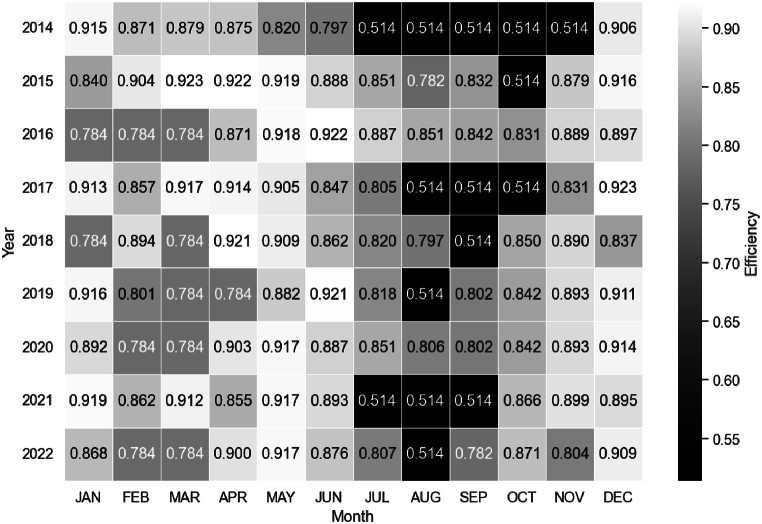
Simulated turbine efficiency for the operating period from 2014 to 2022.

Fig. 8 shows the heat map graph of the simulated turbine efficiency from 2014 to 2022, obtained from Equation (18). The efficiency exhibits a seasonal pattern, with the highest values occurring in the first half of the year and in December. Notably, August shows a significant drop in efficiency in most of the considered years. There is a monthly variation in efficiency, but the general pattern remains consistent.

**Fig. 9:**
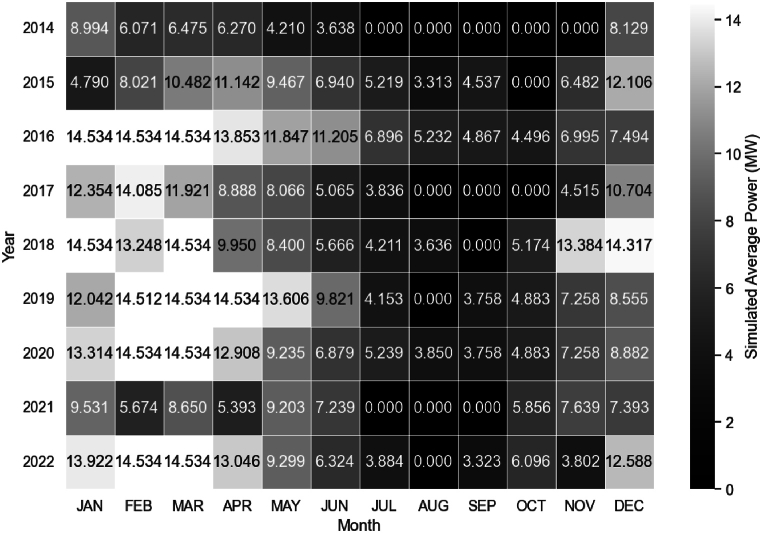
Simulated Average Power for the operating period from 2014 to 2022.

Fig. 9 shows the heat map graph of the simulated average power for the operating period between 2014 and 2022. The months with no power generation correspond to the period when the minimum turbine flow was not reached. The months of December and the first five months of the year exhibit the highest power generation.

**Fig. 10:**
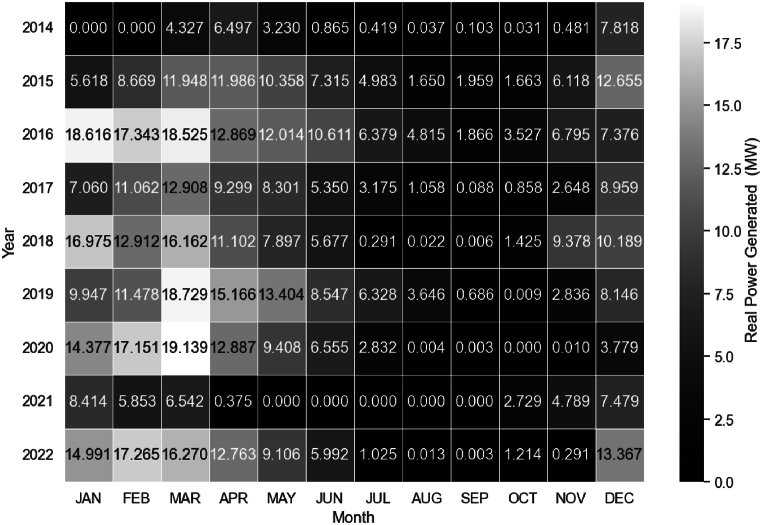
Real Average Power generated at the Retiro SPPH, for the operating period from 2014 to 2022.

Fig. 10 shows the real power generated at the Retiro SHPP. The first year of operation saw very low power generation. The maximum power of 7.8 MW was reached in December 2014. There was no power generation in October 2020, and there were five consecutive months of zero generation in 2021. The low water flow available in the Sapucaí River, combined with the pandemic period, may have contributed to this. Although the SHPP has an installed capacity of 16 MW, in some years of operation, higher power values were recorded during the months of January, February, and March. This can be attributed to the fact that synchronous generators are designed to achieve up to 10 % more power beyond the established limit. Typically, SHPP operators choose to perform maintenance during the months of August or September, as they are periods of the year with lower flow rates in the Sapucaí River. The decrease in energy production during the latter half of the year may be attributed to maintenance activities or other operational interruptions.

**Fig. 11:**
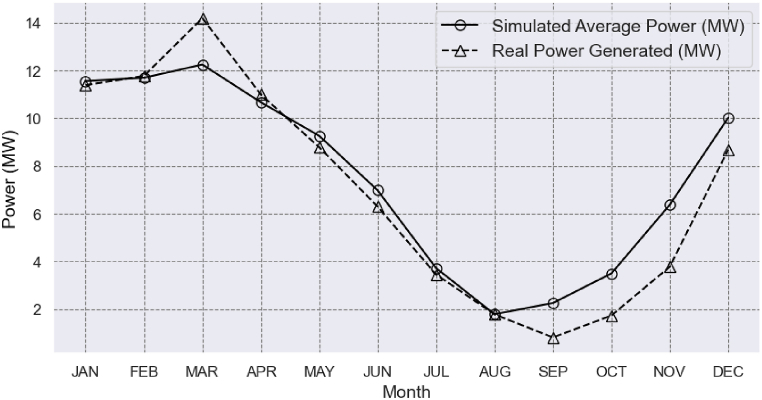
Comparison between the Real Average Power generated at the Retiro Small Hydroelectric Power Plant and the Simulated Average Power for the years 2014–2022.

Fig. 11 shows the correlation between the actual average power generated at the Retiro Small Hydroelectric Power Plant (SHPP) from 2014 to 2022 and the monthly average power simulated during the same period, accounting for losses in the hydraulic circuit. The real power series was provided by the unit operator and recorded by the Brazilian Chamber of Electric Energy Commercialization (CCEE), which oversees energy trading in Brazil. The highest actual average powers are produced in the first half of the year and in December, with March standing out for its potential of 14.19 MW. The months from July to November are the most critical periods for power generation at Retiro SHPP. For example, in September, when the SHPP has the lowest available flow rates, the potential was only 0.81 MW. Operational efficiency during this period is a concern for operators.

These power generation data can be compared with the calculated power data in this study. Overall, the power data followed the same monthly trend. The actual power values were very close to the simulated power values in January, March, April, May, June, July, and August. However, in September, October, and December, the simulated values were approximately 1.5 MW higher.

## 4 Conclusions

The pressure drop in the system depends not only on the outflow but also on the physical conditions of the hydraulic circuit of the Small Hydroelectric Power Plant (SHPP). This study reveals the variability of hydraulic losses over the historical series from 1990 to 2022 based on the hydraulic circuit settings of the Retiro Small Hydroelectric Power Plant. As this is a straightforward configuration project, its load loss was concentrated on the sediment retention grid, at the entrance to the water intake and at the inlet to the turbine. In the analysis of head losses in the SHPP, other elements such as penstocks, pipes, bends, etc., can also be considered.

The study, through normalized data of head losses and flow in the hydraulic system of the Small Hydroelectric Plant (SHPP), demonstrated a gradual reduction in generated power in the second half of the year. A strong correlation was identified between observed flows and head losses.

In the first four months of the year, the SHPP recorded significant losses in relation to electricity generation. March had the highest average powers and experienced the greatest head losses and the best power utilization. In September, occurred the lowest energy production occurred. Given this scenario, it is crucial to pay special attention to the operation of the SHPP during the period from July to October. Considering that this period characterizes the lowest head losses and considerable availability of water resources, it presents a valuable opportunity to maximize energy generation. The results demonstrate that head losses affect the overall efficiency of energy conversion in the SHPP, as observed through the reduction in effectively produced power.

Upon analysis of Fig. 11, it becomes evident that methodology of this study accurately reflects reality, particularly during periods of high flow. The simulated average power, including load losses, closely approximates the actual average power produced at the SHPP in January, February, April, May, June, July, and August. However, the simulated power did not correspond as closely during the months of September, October, November, December, and March, despite exhibiting the same trend. It is possible that other factors, such as electricity prices, the maintenance period of the SHPP, and turbine efficiency in relation to water flow availability, will have influenced the actual power production, which were not considered in this study.

The SHPP Retiro has only one electricity-generating unit. Annually, usually a period for predictive maintenance is scheduled for the month of September, which is a period of lower water flow. This scheduled unavailability contributes to the reduction of effectively produced energy in that month, thus wasting a valuable opportunity to generate more energy. Considering the hypothesis that the Small Hydroelectric Power Plant (SHPP) had two generating units with a power of eight MW each, the plant operator could schedule predictive maintenance for each generating unit separately in the months of August and September or September and October, maximizing the use of water resources during this period. While one unit is undergoing maintenance, the other would be generating electrical energy. These suggestions imply a more in-depth study of economic feasibility. Furthermore, the authors recommend a more detailed investigation into the impact of daily power on the hydraulic circuit. It is also suggested to improve other specific areas of the circuit where load losses may occur.

## Data availability statement

Data included in article/supp. material/referenced in article.

## Ethics declarations

Review and/or approval by an ethics committee were not needed for this study because it does not involve human subjects, interviews, questionnaires etc. The study in this article utilizes data provided by the National Electric Energy Agency - Brazil (ANEEL). The data is available for public consultation.

## CRediT authorship contribution statement

**Francisco Wellington Martins da Silva:** Writing – original draft, Software, Resources, Project administration, Methodology, Investigation, Data curation, Conceptualization. **José Roberto Camacho:** Writing – review & editing, Validation, Supervision, Resources, Funding acquisition, Formal analysis, Conceptualization. **Jacson Hudson InácioFerreira:** Writing – original draft, Data curation, Conceptualization, Supervision.

## Declaration of competing interest

The authors declare the following financial interests/personal relationships which may be considered as potential competing interests: Francisco Wellington Martins da Silva reports a relationship with Coordination for the Improvement of Higher Education Personnel - Brazil (CAPES) that includes: funding grants. If there are other authors, they declare that they have no known competing financial interests or personal relationships that could have appeared to influence the work reported in this paper.
